# Can General Practitioner Opioid Prescribing to Compensated Workers with Low Back Pain Be Detected Using Administrative Payments Data? An Exploratory Study

**DOI:** 10.1007/s10926-024-10194-y

**Published:** 2024-04-02

**Authors:** Jennifer Vo, Shannon Gray, Adrian C. Traeger, Michael Di Donato

**Affiliations:** 1 Healthy Working Lives Research Group, School of Public Health and Preventive Medicine, Monash University, 553 St Kilda Road, Melbourne, VIC 3004 Australia; 2 Institute for Musculoskeletal Health, School of Public Health, The University of Sydney, Camperdown, NSW Australia

**Keywords:** Low back pain, Workers’ compensation, Administrative data methods

## Abstract

**Background:**

Approximately one third of Australians with accepted time loss workers’ compensation claims for low back pain (LBP) are dispensed opioid analgesics. Structured administrative payments data is scalable but does not directly link opioids to prescribers. We sought to determine whether opioid prescribing by general practitioners (GPs) to workers with workers’ compensation claims for LBP can be detected in structured administrative payments data.

**Methods:**

We used a sample of workers with accepted time loss workers’ compensation claims for low back pain from 2011 to 2015 from the Australian states of Victoria and South Australia. We structured administrative data to test the assumption that opioid dispenses that occurred immediately after a GP encounter in sequence and occurred on the same date as the GP encounter are likely to be related. We measured the number and proportion of opioid dispenses with a GP encounter prior and the days between a GP encounter and opioid dispense.

**Results:**

Nearly one third of workers (32.2%, *N* = 4,128) in our sample (*n* = 12,816) were dispensed opioids a median of five times (interquartile range 2, 17). There were 43,324 opioid dispenses to included workers. 30,263 (69.9%) of opioid dispenses were immediately preceded by a GP encounter. Of those dispenses, 51.0% (*n* = 15,443) occurred on the same day as the GP encounter.

**Conclusion:**

At least one third of opioids dispensed to workers with claims for LBP can be potentially linked to GP prescribing using workers’ compensation structured administrative payments data. This approach could have potential applications in supporting monitoring and audit and feedback systems. Future research should test this approach with a more diverse array of pain medicines and medical practitioners.

**Supplementary Information:**

The online version contains supplementary material available at 10.1007/s10926-024-10194-y.

## Background

Recent guidelines and research highlight that the potential limited benefits of opioids for managing low back pain may be outweighed by harmful side effects [1–3]. In many countries, opioid analgesics usually require a prescription. General practitioners (GPs, i.e., general physicians or family physicians) are the primary care provider for most health concerns and medicines prescribing, including for low back pain. In Australia, back pain is one of the most frequent presentations to general practitioners [4]. Furthermore, a cross-sectional survey of 3897 general practitioners between 2006 and 16 estimated nearly 40% of back problems presenting to Australian general practitioners resulted in an opioid prescription [5].

People unable to work due to low back pain that developed during their employment may be eligible for income replacement and funding for health care from a workers’ compensation system. Time loss claims for low back pain are relatively common in Australian workers’ compensation schemes [6]. Furthermore, approximately one third of workers with low back pain are dispensed opioids at some point in their claim [7]. General practitioners are the primary health care provider and point of contact in most workers’ compensation schemes; they are often responsible for managing return to work, ongoing certification of capacity and referral to other health care providers in addition to providing health care themselves [8]. It is, therefore, likely that the most common prescriber of opioids for compensated workers with low back pain are general practitioners, although there is limited research to support this theory.

Workers’ compensation schemes generate large volumes of data through normal operations (i.e., managing claims and tracking expenditure). This includes rich unstructured data in the form of case notes made by claims managers, and structured administrative data such as itemised service payment records [9]. Information about general practitioner opioid prescribing likely exists in unstructured data such as case notes and communications between claims managers and general practitioners. However, this would be challenging to scale to a broader monitoring or audit and feedback system that could identify trends in prescribing. Structured administrative data could enable such a system; however, it is unclear whether payments for general practitioner services and subsequent purchases of opioids can be readily linked via payments data. In this exploratory study we sought to determine whether opioid prescribing by general practitioners to workers with workers’ compensation claims for low back pain can be detected in structured administrative payments data.

## Methods

### Setting

There are workers’ compensation schemes in each Australian state and territory, and three national schemes. Each scheme has different policies and practices, but all ultimately provide funding for income replacement and reasonable and necessary health care to support worker recovery. This study was conducted using data from the Australian states of Victoria and South Australia, with a labour force of approximately 3.8 million persons in 2015 [10]. At the time of the study, both schemes required employers fund the first two weeks of income replacement. The Victorian scheme requires employers also fund the first $700 (AUD) of health care [11].

### Data Source

We used a sample from the Multi-Jurisdiction Workers’ Compensation Database [9]. This database contains structured claims (e.g., worker age, sex, occupation) and payments data (e.g., health care type, date, cost) from five Australian states and territories. We included data only from Victoria and South Australia because the workers’ compensation authorities in these states collect detailed data about medicines. Medicines data were matched to Anatomical Therapeutic Chemical (ATC) codes and general practitioner (GP) services were identified in processes described elsewhere [9]. In brief, once a prescription has been provided in a consultation with a GP or other medical practitioner, it is purchased from a pharmacy and the cost claimed back from the workers’ compensation system. Opioids were identified via ATC level 5 codes, listed in the supplementary materials.

### Sample

We included workers with accepted time loss workers’ compensation claims for low back pain between July 2011 and June 2015 (i.e., four Australian financial years). Low back pain was defined using the Type of Occurrence Classification System (TOOCS) (see supplementary materials). Workers were aged between 15 and 80 years. We included any GP encounter and opioid dispense to workers 30 days before to 730 days (i.e., two years) after the claim acceptance date. Services may be retrospectively reimbursed, hence the 30 days prior to claim acceptance. Two years is a relatively standard follow-up period used in previous studies. Duplicated records of services or medicines (e.g., two opioid dispenses on the same date) were removed, as we only sought to explore coinciding general practitioner encounters and opioid prescriptions, not necessarily the amount or dose of opioid prescriptions.

### Outcome Measures

We measured the number and proportion of opioid dispenses with a GP encounter in sequence before an opioid dispense (i.e., appearing in administrative data before an opioid dispense), and the number of days between GP encounter and opioid dispense.

### Analysis

Our assumption was that opioid dispenses with a GP encounter immediately prior in sequence (i.e., not two or more opioids dispensed sequentially without GP encounter prior), that occurred on the same date as the GP encounter, are likely to be related. To test this assumption, we prepared the data in a specific way and measured the proportion of opioid dispenses that met our assumption.

Administrative health care services and medicines datasets were structured with the same three critical variables: a worker identifier, date of service (or dispense in the case of opioids), and the type of service (e.g., GP encounter, N02AA05 Oxycodone, etc.). We first simplified both datasets to make the type of service a binary indicator: GP encounter or opioid dispense. We then appended the two datasets. We calculated the days between claim acceptance and each service. Data were ordered by the worker identifier and the date of service / dispense. We created a “lag” variable that reported the service and days to that service in the previous row. We then created a second lag variable that reported the service and days to service two rows earlier. We created these sets of variables for up to 10 rows for each worker. This data structure allowed us to identify in what sequence and when a GP encounter occurred relative to an opioid dispense.

**Fig. 1:**
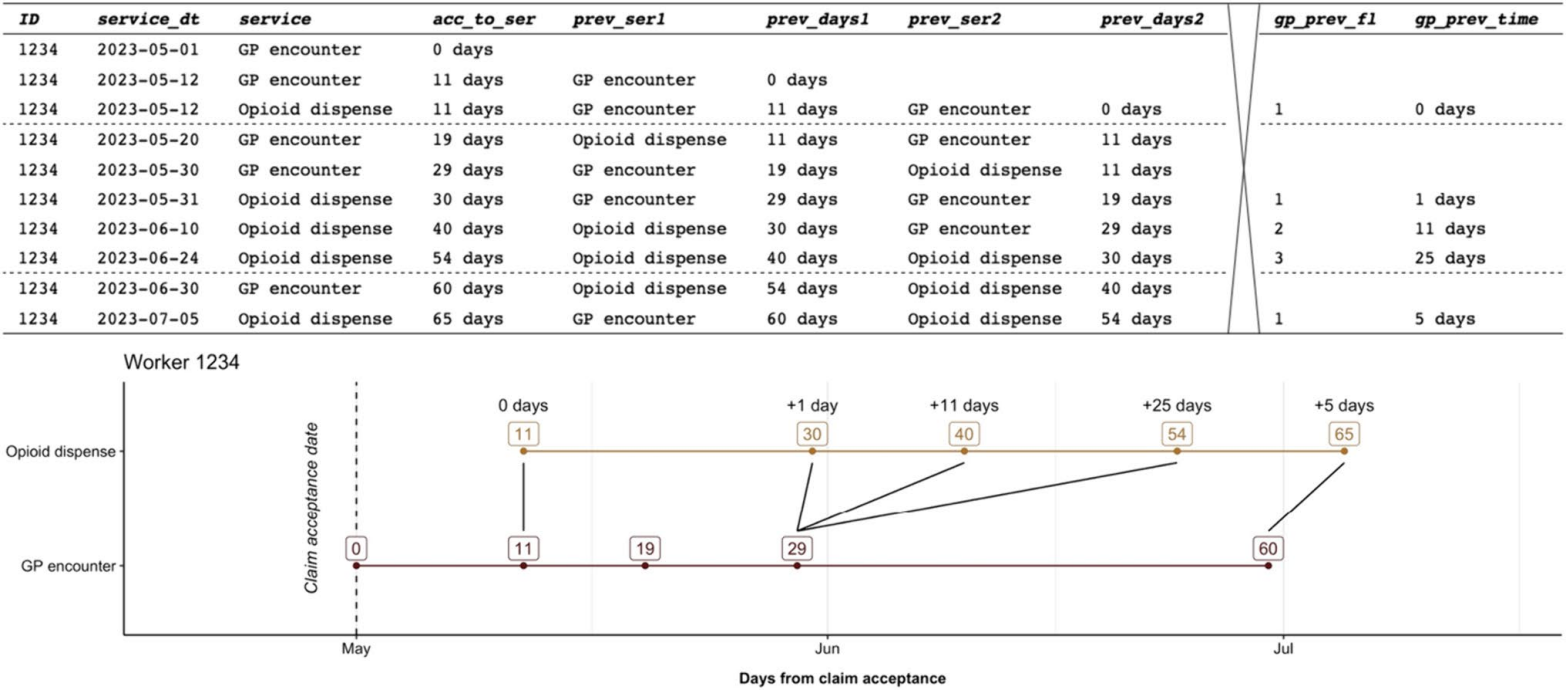
Example of data structure for a single worker. Numbers in boxes represent the number of days from claim acceptance to GP encounters and opioid dispenses

An example of how data were structured is available in Fig. 1. In this example, a worker is dispensed opioids five times and encounters a GP five times. The worker is first dispensed opioids 11 days from claim acceptance. A GP encounter is the service immediately prior in sequence (i.e., appearing in administrative data before an opioid dispense) and occurs on the same date. The worker is dispensed opioids a number of times later in their claim, but these occur in different sequence and days from a GP encounter.

We used descriptive statistics to report the number and percentage of opioid dispenses that had a GP encounter one row before (i.e., immediately prior in sequence) and two through up to more than ten rows before. We then selected only opioid dispenses with a GP encounter immediately prior in sequence and measured the number and percentage that occurred on each of zero (i.e., the same day) through to 14 days from the GP encounter.

Analyses were conducted in IBM SPSS and R 4.2.2. Ethics approval was provided by the Monash University Human Research Ethics Committee (Project ID 17267, November 2018).

## Results

We included 12,816 workers with accepted time loss workers’ compensation claims for low back pain from Victoria (72.6%, *n* = 9,305) and South Australia (27.4%, *n* = 3511). Most workers encountered a GP (81.7%, *n* = 10,475) a median of 11 times (interquartile range (IQR) 4, 25). Nearly one third of workers (32.2%, *n* = 4,128) were dispensed opioids a median of five times (IQR 2, 17). Further sociodemographic information about the sample is available in the supplementary materials.

**Fig. 2:**
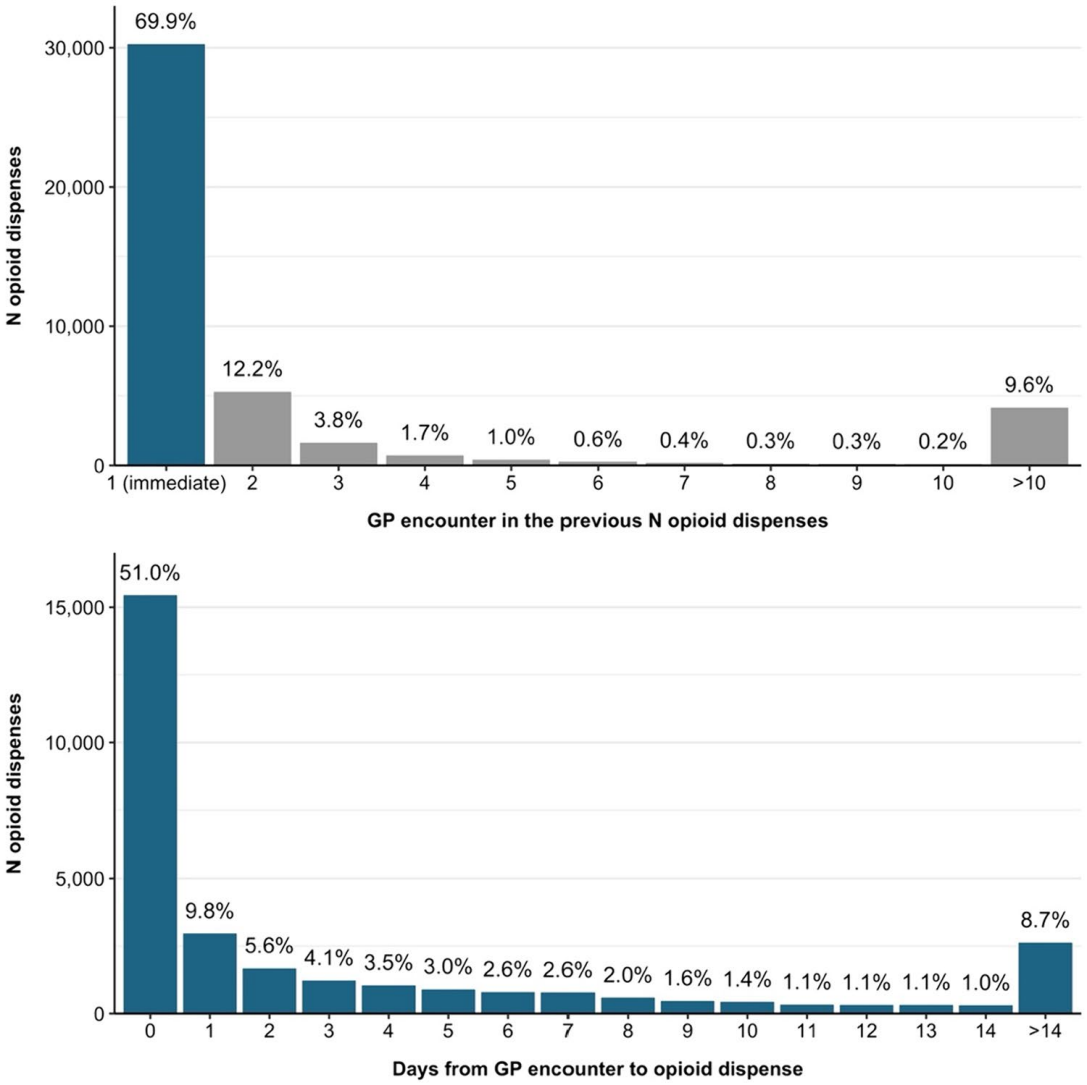
Top chart: the N of opioid dispenses that had a GP encounter immediately prior in sequence, 2 previous, 3 previous, and so on. Bottom chart: N of opioid dispenses by the number of days between GP encounter and opioid dispense

There were 43,324 opioid dispenses to included workers. Nearly 70% of opioid dispenses had a GP encounter immediately prior (69.9%, *n* = 30,263), with 12.2% (*n* = 5305) having a GP encounter two services prior (see Fig. 2). Approximately half of the opioid dispenses with a GP encounter immediately prior in sequence had a GP encounter zero days from the opioid dispense (51.0%, *n* = 15,443). We can, therefore, assume that at least 35.6% (*n* = 15,443) of all opioids dispensed were prescribed by a GP.

## Discussion

In this exploratory study we identified that at least one third of opioid dispenses can be potentially linked to general practitioner prescribing using workers’ compensation structured administrative payment data. Most opioid dispenses are immediately preceded by a general practitioner encounter, and of those, half occurred on the same day. Although we cannot directly link the two services, we believe that this exploratory analysis supports our assumption that opioid dispenses occurring immediately afterwards in sequence and on the same date as general practitioner encounters can be associated.

General practitioners are central to workers’ compensation and medicines prescribing. From this exploratory research, it appears that general practitioners may be a common opioid prescriber for workers with low back pain in the workers’ compensation sector. In reality it appears that a third of dispenses are acquired on the same date, likely directly after, a general practitioner encounter – in which the worker probably obtained a script. Other opioid dispenses without a general practitioner encounter immediately prior (30.1%) might have been a repeat script or prescribed by a different medical practitioner.

Prescription drug monitoring programs (PDMP) to support clinical decision making have been deployed in both included jurisdictions since the study period [12, 13]. However, linking general practitioners to opioid dispensing using this method could support more focused monitoring or audit and feedback interventions to reduce opioid prescribing for low back pain. This has been attempted with diagnostic imaging referrals by general practitioners in a previous study [14]. Our proposed method of matching opioid dispenses to services is not complex and, using aggregated output, does not risk re-identification or pose privacy concerns. General practitioners could be provided with their rate of opioid dispensation for workers with back pain relative to their peers. One alternative method may be to simply identify the proportion of compensated workers consulted by a given general practitioner who are dispensed opioids. Although the opioids could be prescribed from other sources, this may be a useful way to notify general practitioners whether more or less of their patients are dispensed opioids relative to a larger average.

The method proposed in our paper is not necessarily limited to a single health care system. From a clinical perspective, PDMPs already offer a reliable option to monitor opioid prescriptions. Our proposed method would be best suited to health care funders or insurers, who have a broad scope across all aspects of an individuals’ health care. Workers’ compensation schemes, who fund all reasonable and necessary aspects of injured worker health care, are one example of a system best suited to utilise this method. As described above, funders could apply the method to existing administrative payments data to produce benchmarks or conduct routine monitoring.

While our study benefited from a large sample size and novel approach, we also acknowledge several limitations. Firstly, our sample was relatively limited, including only those with workers’ compensation claims for low back pain and those dispensed opioids. Secondly, we only reported that an opioid dispense had occurred, neglecting information about type, strength or dose of opioids. Most notably, we did not include any of the many other medical practitioner specialisations who can prescribe opioids, such as surgeons, pain specialists or occupational physicians. While general practitioners are the primary health care provider, we cannot ignore the strong possibility that other health care providers prescribed opioids. Finally, our data only includes medicines funded by the workers’ compensation scheme. With relatively accessible health care in Australia, it is possible that workers may have obtained opioids funded by other sources, such as out-of-pocket expenses or private health insurance.

Future research would benefit from two important inclusions. Firstly, any follow-up study should include all pain medicines prescribed to workers. While opioids have been a priority concern for many years, recent literature has highlighted the increasing off-label use of medicines such as gabapentinoids and antidepressants for low back pain [15–17]. Additional information about pain medicines, such as multiple prescriptions, strength, dose and duration, would also be beneficial. Secondly, future research should sequence any and all services relative to pain medicine dispenses. Our approach could then be applied backwards, by identifying the health care services that occurred in eligible sequential and time proximity to a pain medicine dispense.

## Supplementary Information

Below is the link to the electronic supplementary material.

Supplementary file1 (PDF 184 KB)

## Data Availability

Data used in this paper are not available for distribution by the authors.
