# Supplementary material for: Can General Practitioner Opioid Prescribing to Compensated Workers with Low Back Pain Be Detected Using Administrative Payments Data? An Exploratory Study
Source: J Occup Rehabil. 2024 Apr 2;35(1):48–53. doi: 10.1007/s10926-024-10194-y (PMC11839698; doi:10.1007/s10926-024-10194-y)
Supplement: Supplementary file 1 — Supplementary file1 (PDF 184 KB) [file 10926_2024_10194_MOESM1_ESM.pdf]

Can general practitioner opioid prescribing to compensated workers with low back pain be detected using administrative payments data? An exploratory study

Jennifer Vo[1], Shannon Gray[1], Adrian C Traeger[2], and Michael Di Donato[1]

1. Healthy Working Lives Research Group, School of Public Health and Preventive Medicine, Monash University
2. Institute for Musculoskeletal Health, School of Public Health, The University of Sydney

Corresponding author

Dr Michael Di Donato

Healthy Working Lives Research Group, School of Public Health and Preventive Medicine, Monash University

553 St Kilda Road, Melbourne, VIC 3004, Australia

E: michael.didonato@monash.edu

T: +61 9905 6417

SUPPLEMENTARY MATERIALS

Supplementary Table 1. Included opioids

ATC Code	ATC Description	Opioid Strength
N02AJ06	Codeine and paracetamol	Weak
N02AJ07	Codeine and acetylsalicylic acid	Weak
N02AJ08	Codeine and ibuprofen	Weak
N02AJ13	Tramadol and paracetamol	Weak
N02AX02	Tramadol	Weak
N02AC54	Dextropropoxyphene, combinations excl. psycholeptics	Weak
N02AC04	Dextropropoxyphene	Weak
N02AA01	Morphine	Strong
N02AA03	Hydromorphone	Strong
N02AA05	Oxycodone	Strong
N02AA55	Oxycodone and naloxone	Strong
N02AB02	Pethidine	Strong
N02AB03	Fentanyl	Strong
N02AE01	Buprenorphine	Strong
N02AX06	Tapentadol	Strong

Opioid classification sourced from:

1. *Dowell D, Haegerich TM, Chou R. CDC Guideline for Prescribing Opioids for Chronic Pain. MMWR Recomm Rep. 2016;65(No. RR-1):1-49.*
2. *Mathieson S, Wertheimer G, Maher CG, Christine Lin CW, McLachlan AJ, Buchbinder R, et al. What proportion of patients with chronic noncancer pain are prescribed an opioid medicine? Systematic review and meta-regression of observational studies. J Intern Med. 2020;287(5):458-74.*

Supplementary Table 2. Eligible TOOCS codes

TOOCS Parameter	Low Back Pain
Nature of Injury	228 – Trauma to muscles and tendons, not elsewhere classified<br>229 – Trauma to muscles and tendons, unspecified<br>239 – Soft tissue injuries due to trauma or unknown mechanisms with insufficient information to code elsewhere<br>422 – Disc displacement, prolapse, degeneration, or hernia<br>459 – Back pain, lumbago, and sciatica<br>533 – Muscle / tendon strain (non-traumatic)
Bodily Location of Injury	311 – Lower back
Mechanism of Injury	Any
Agency of Injury	Any

Supplementary Table 3. Descriptive statistics

	Opioid Dispenses		***GP Encounters***		***Total***
	N (row %)	*Median (IQR)*	*N (row %)*	*Median (IQR)*	*N*
Whole sample	4,128 (32.2)	5 (2, 17)	10,475 (81.7)	11 (4, 25)	12,816
Sex					
Female	1,396 (31.3)	5 (2, 17)	3,660 (82.0)	11 (5, 25)	4,464
Male	2,732 (32.7)	5 (2, 17)	6,815 (81.6)	11 (4, 26)	8,352
Age Group					
15-25 years	288 (19.7)	3 (1, 10)	1,117 (76.3)	8 (3, 17)	1,463
26-35 years	916 (31.3)	5 (2, 17)	2,377 (81.3)	10 (4, 23)	2,925
36-45 years	1,195 (36.3)	6 (2, 22)	2,763 (83.9)	12 (5, 27)	3,295
46-55 years	1,178 (34.7)	5 (2, 17)	2,780 (82.0)	12 (5, 27)	3,390
56+ years	551 (31.6)	5 (2, 14)	1,438 (82.5)	11 (5, 25)	1,743
Occupation					
Clerical and Administrative Workers	105 (35.6)	7 (2, 20)	242 (82.0)	11 (4, 23)	295
Community and Personal Service Workers	647 (28.3)	5 (2, 16)	1,837 (80.4)	10 (4, 23)	2,284
Labourers	1,023 (31.0)	5 (2, 16)	2,654 (80.4)	11 (4, 26)	3,300
Machinery Operators and Drivers	837 (34.1)	5 (2, 20)	2,061 (83.8)	12 (5, 27)	2,458
Managers	250 (38.3)	5 (2, 21)	541 (82.8)	12 (5, 26)	653
Professionals	309 (32.5)	5 (2, 16)	736 (77.5)	10 (4, 23)	950
Sales Workers	189 (35.5)	5 (2, 15)	449 (84.4)	12 (5, 26)	532
Technicians and Trades Workers	767 (32.7)	5 (2, 17)	1,954 (83.4)	10 (4, 24)	2,343
Jurisdiction					
Victoria	2,849 (30.6)	6 (2, 21)	7,095 (76.2)	12 (5, 27)	9,305
South Australia	1,279 (36.4)	3 (1, 10)	3,380 (96.3)	9 (4, 21)	3,511
Socioeconomic Status					
Most advantaged quintile	527 (28.7)	4 (1, 14)	1,412 (76.8)	10 (4, 23)	1,838
Second to fourth quintiles	2,603 (32.1)	5 (2, 18)	6,598 (81.4)	11 (4, 25)	8,108
Most disadvantaged quintile	813 (33.7)	6 (2, 19)	2,034 (84.2)	11 (5, 27)	2,415
Missing (n=455)	185 (40.7)	5 (2, 15)	431 (94.7)	12 (5, 26)	455
Remoteness					
Major Cities of Australia	2,838 (31.9)	5 (2, 17)	7,233 (81.3)	11 (5, 26)	8,900
Regional Australia	1,077 (32.0)	6 (2, 19)	2,724 (81.0)	10 (4, 24)	3,364
Remote Australia	24 (27.3)	4 (2, 14)	80 (90.9)	6 (3, 12)	88
Missing (n=464)	189 (40.7)	5 (2, 15)	438 (94.4)	12 (5, 26)	464
